# An Interesting Case of Ocular Nocardiosis Mistaken as a Fungal Corneal Ulcer

**DOI:** 10.7759/cureus.96672

**Published:** 2025-11-12

**Authors:** Sharah Rahman, Ishtiaque Anwar, Tarzia Asma Zafrullah, Jalal Ahmed, Shahrina Mahfooz

**Affiliations:** 1 Cornea and Refractive Surgery, Bangladesh Eye Hospital and Institute, Dhaka, BGD; 2 Ophthalmology, Bangladesh Eye Hospital and Institute, Dhaka, BGD; 3 Ophthalmology, Lions Eye Institute &amp; Hospital, Dhaka, BGD; 4 Cataract, Refractive Surgery, Glaucoma and Anterior Segment, Bangladesh Eye Hospital and Institute, Dhaka, BGD; 5 Pediatric Ophthalmology and Strabismus, Bangladesh Eye Hospital and Institute, Dhaka, BGD

**Keywords:** agricultural trauma, amikacin, corneal ulcer, fungal keratitis, fungal mimic, gram-positive filament, infectious keratitis, nocardia keratitis, ocular nocardiosis

## Abstract

Ocular nocardiosis is a rare corneal infection that often mimics fungal keratitis, resulting in diagnostic uncertainty and potential delays in appropriate treatment. We describe the case of a 47-year-old woman from a rural farming community who developed a painful, red eye after agricultural trauma. She had been treated empirically for presumed fungal keratitis with topical natamycin and clotrimazole, along with oral ketoconazole for one month, yet her symptoms progressed. On presentation, her vision was limited to hand movements near the face. Slit-lamp examination revealed a wreath-shaped suppurative stromal and epithelial infiltration with feathery branches of the ulcer and a two-millimetre hypopyon with marked corneal thinning, without satellite lesions. Corneal scrapings were examined using potassium hydroxide (KOH) and Gram stain. The KOH mount showed no fungal filaments, whereas the Gram stain revealed thin, beaded, branching, Gram-positive filaments suggestive of Nocardia species. Antifungal therapy was discontinued, and treatment with topical moxifloxacin and amikacin 2% hourly, bedtime tobramycin ointment, and oral moxifloxacin was initiated. Clinical and symptomatic improvement occurred dramatically within one week, and the ulcer healed over the next month, leaving a faint scar. The best-corrected vision was improved to 6/18 (pinhole). The residual blur was due to astigmatism from corneal irregularity. This report highlights how nocardial keratitis may imitate a fungal corneal ulcer clinically, and stresses that early laboratory confirmation enables targeted antibiotic therapy, preventing irreversible visual loss.

## Introduction

The Nocardia species are filamentous, mildly acid-fast, aerobic bacteria typically found in soil and decomposing vegetation. When agricultural ocular trauma introduces the organism into the cornea, it can cause infectious keratitis, particularly among individuals engaged in agricultural work or outdoor activities [1]. Individuals from low socioeconomic backgrounds and underdeveloped communities are more susceptible to nocardial infections due to limited access to healthcare and poor nutritional status. Other ocular manifestations of nocardial infection include scleritis, conjunctivitis, orbital cellulitis, and endophthalmitis, often occurring secondary to ocular trauma. Less common predisposing factors include prior ocular surgery, use of topical corticosteroids, and contact lens wear [2]. A detailed history, including prior ocular infections and ophthalmic consultations, is essential, as previous inflammatory or infectious episodes may have contributed to the development of nocardial keratitis. The lesion often appears as a patchy, elevated, chalky-white epithelial and stromal infiltrate with feathery margins, resembling fungal keratitis [3]. Empirical antifungal treatment is therefore frequent and may delay appropriate antibiotic therapy [4]. Unrecognised ocular nocardiosis can cause progressive stromal thinning and corneal perforation, often leading to severe and irreversible visual loss [5]. This case illustrates the diagnostic and therapeutic challenges associated with nocardial keratitis, which was initially misdiagnosed as a fungal corneal ulcer.

## Case presentation

**Figure 1:**
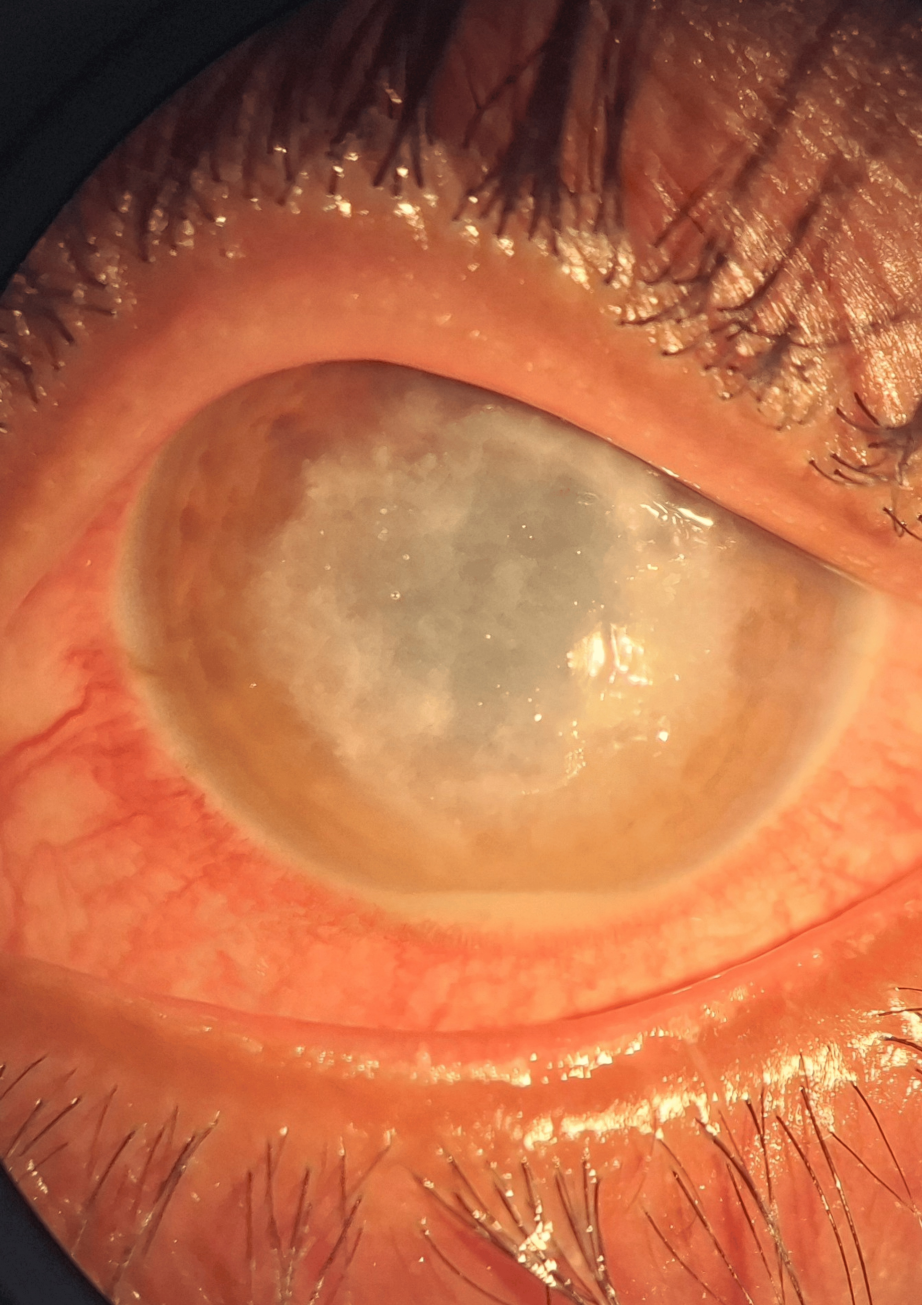
Slit-lamp photographs illustrating a dry, feathery, branching stromal ulcer with a 2 mm hypopyon and marked corneal thinning

A 47-year-old housewife from rural Bangladesh presented with redness, severe pain, photophobia, and diminished vision in her right eye, persisting for one month following exposure to paddy while crossing the field. She had received empirical topical natamycin 5%, clotrimazole drops, and oral ketoconazole for a month at a local clinic without any signs of improvement. On examination, the visual acuity was hand movements close to the face. Slit-lamp examination showed a dense, patchy, greyish white wrath-like stromal infiltrate with fine feathery, branching margins and a 2 mm hypopyon (Figure 1).

**Figure 2:**
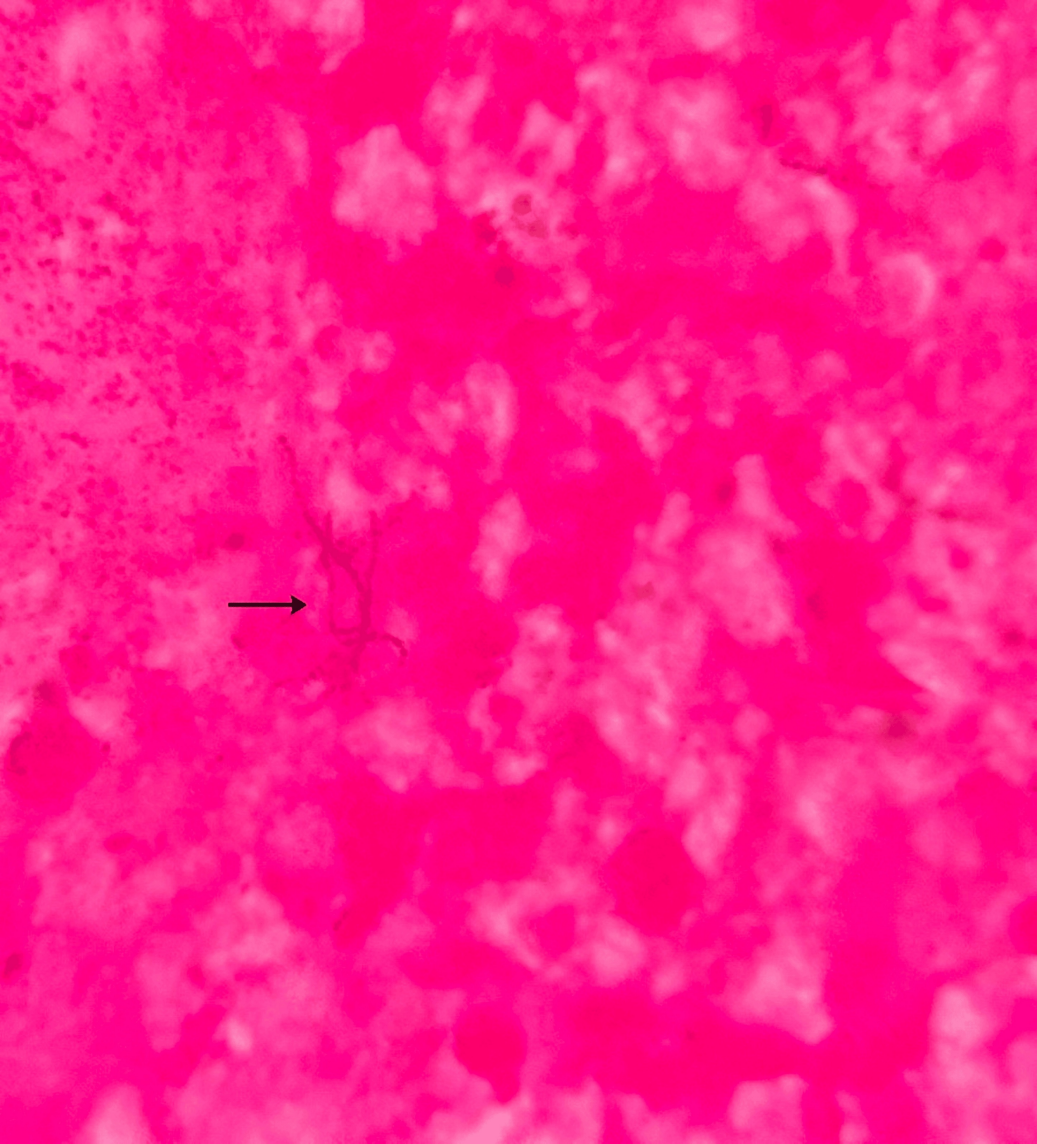
Gram-stained corneal smear showing thin, branching, beaded filamentous Gram-positive organisms compatible with the Nocardia species

**Figure 3:**
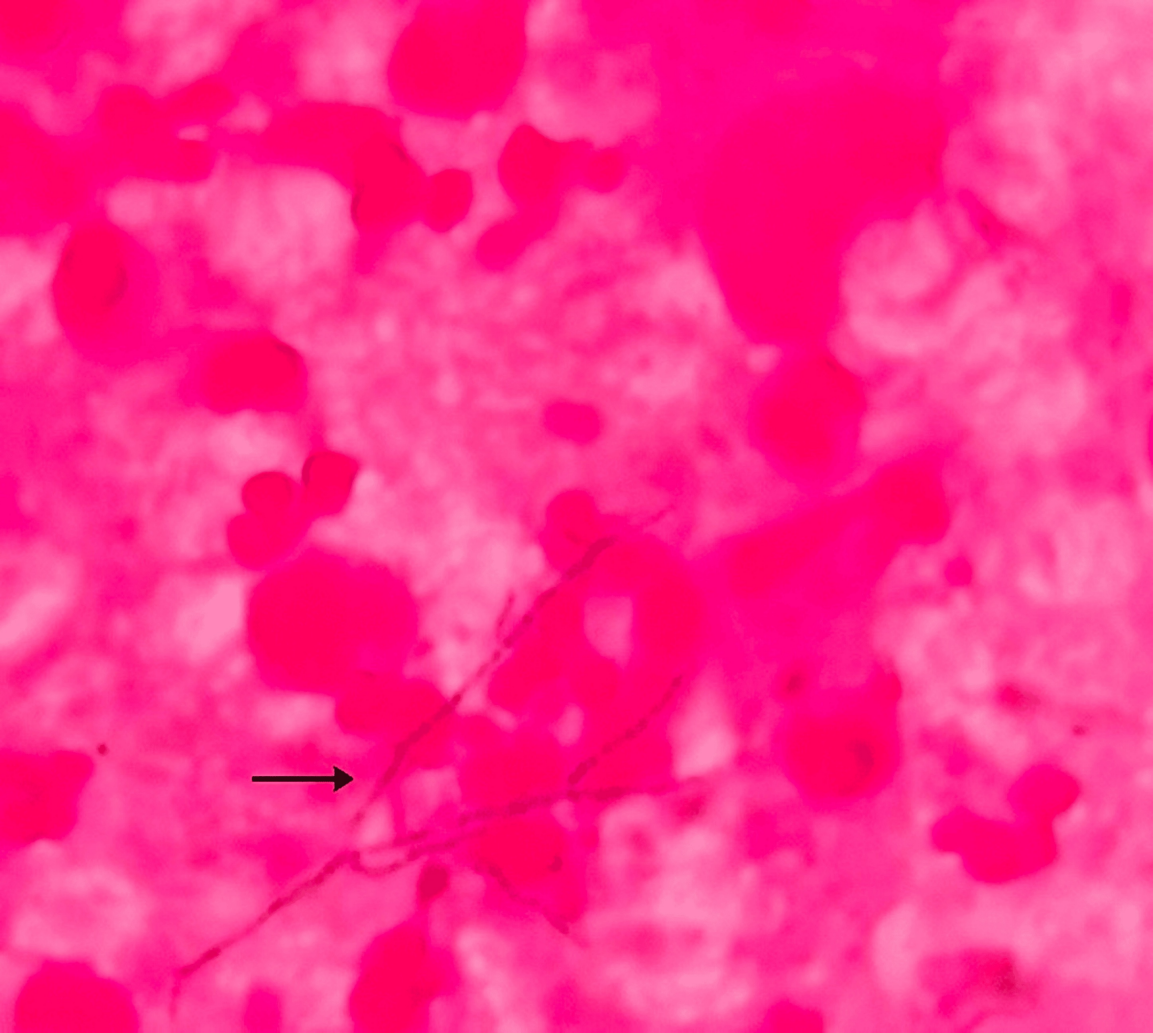
Gram stain of the corneal smear showing typical, branching, beaded Gram-positive organisms compatible with the Nocardia species

The central corneal epithelium was extremely thin, with a chance of impending perforation, but there were no satellite lesions or endothelial plaque. Corneal scrapings showed no fungal elements on the potassium hydroxide (KOH) mount. Gram stain revealed thin, beaded, filamentous Gram-positive organisms, and modified acid-fast stain (1% H_2_SO_4_) identified weakly acid-fast, branching filaments consistent with Nocardia (Figures 2, 3).

**Figure 4:**
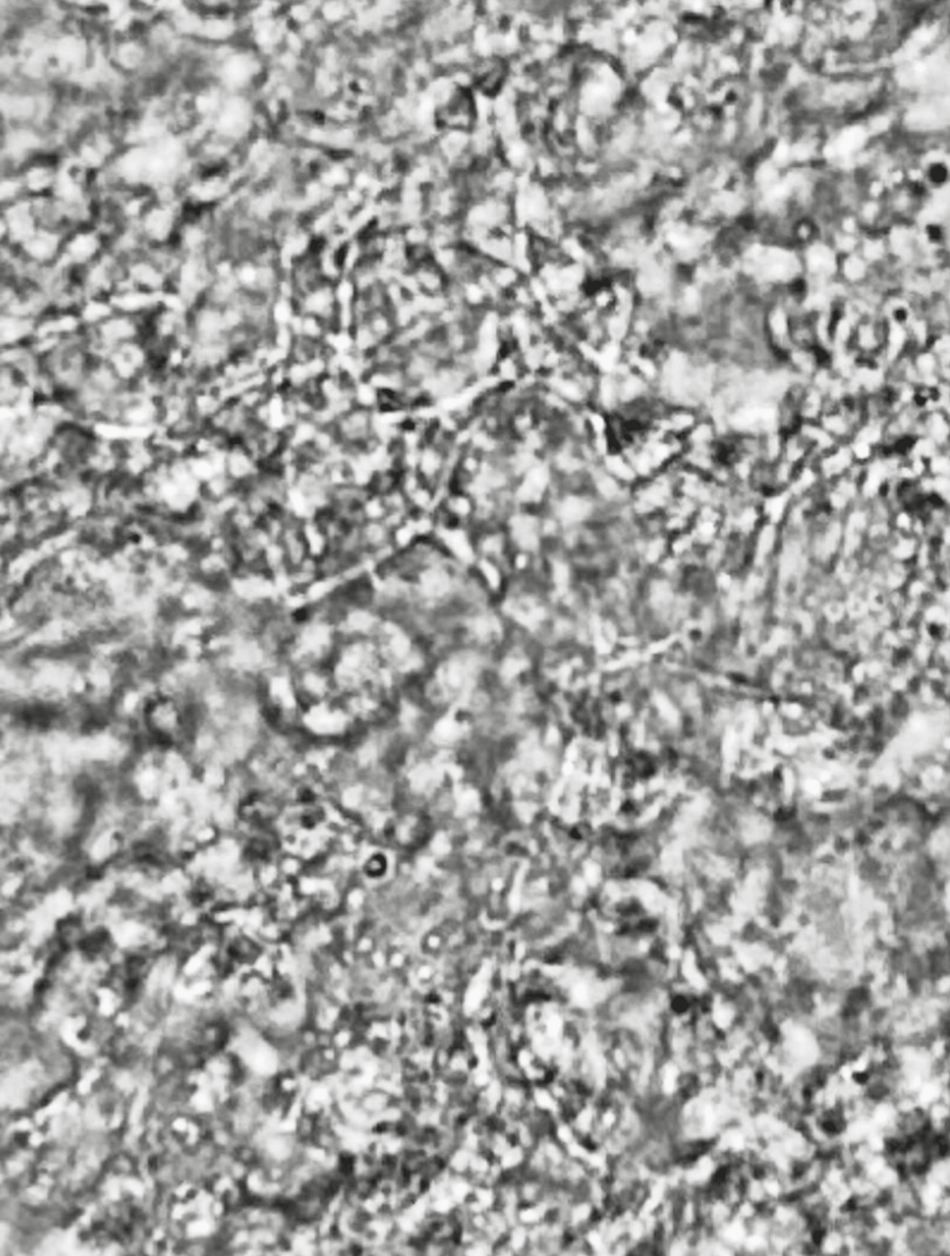
Potassium Hydroxide (KOH) mount smear shows refractile branching pattern of Nocardia under the electron microscope

The KOH mount smear under the electron microscope showed a refractile branching pattern, corresponding with the Nocardia species (Figure 4).

**Figure 5:**
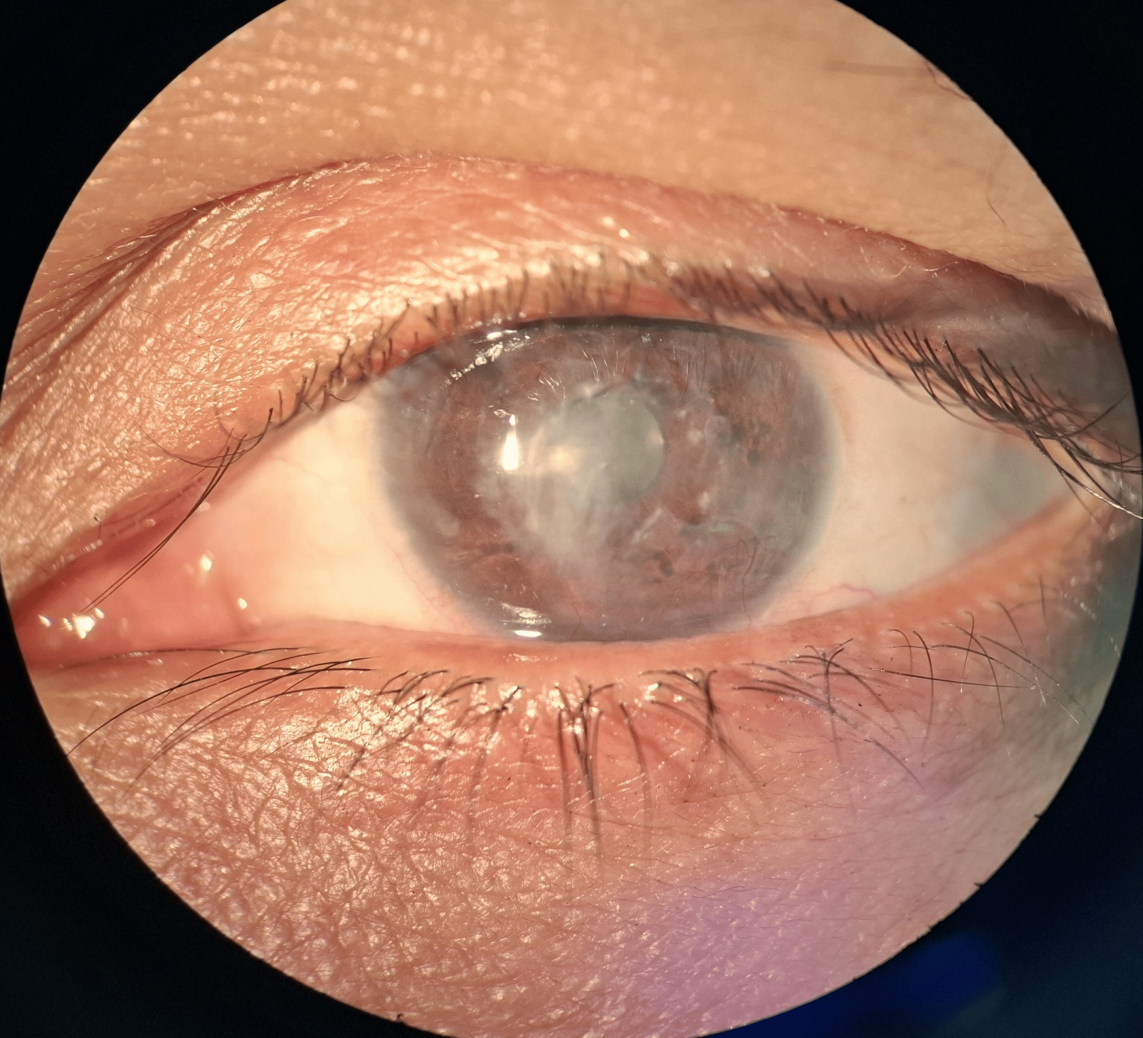
The healed ulcer after three weeks, leaving a small, irregular scar

The culture produced dry, chalk-white colonies, characteristic of the Nocardia keratitis complex. All antifungal medications were stopped immediately. The patient was treated with topical amikacin 2% hourly, moxifloxacin every four hours, tobramycin ointment at bedtime, and oral moxifloxacin 400 mg daily. Dramatic improvement occurred within five days, with reduced pain and clearing of the infiltrate. After three weeks, the ulcer healed, leaving a small, irregular scar (Figure 5).

Her best-corrected visual acuity improved to 6/18. The residual blur was due to corneal astigmatism, which was later managed by Rose K Contact Lens (Menicon Ltd., Nagoya, Japan).

## Discussion

Nocardial keratitis represents a minor category of bacterial corneal diseases found in tropical regions [6]. Because it looks much like fungal ulcers, using empirical antifungal drugs is common among ophthalmologists, which delays the right treatment [7]. We have presented a similar case where Nocardia keratitis mimicked fungal keratitis. Clinically, nocardial keratitis presents as a patchy, elevated granular infiltrate with a delicate branching pattern. Unlike fungal keratitis, satellite lesions are typically absent, and a wreath-like epithelial infiltrate involving stroma may be present [8]. In this patient, persistent symptoms without clinical improvement despite antifungal therapy raised suspicion for an atypical infection. Gram staining, KOH staining, and modified Ziehl-Neelsen staining are essential tests for finding filamentous, weakly acid-fast bacteria [3,9]. Topical amikacin remains the principal treatment due to its highly potent efficacy and good corneal penetration [10]. When deeper involvement is suspected, systemic fluoroquinolones or sulfonamides can be beneficial. Despite one month of inappropriate antifungal therapy, the patient improved dramatically following targeted antibiotic therapy. Residual astigmatism accounted for minor visual limitation, which was later managed by Rose K contact lens. This emphasises the need for routine microbiological validation in all non-healing corneal ulcers [8,11,12].

## Conclusions

Nocardial keratitis should always be considered in non-resolving cases of suspected fungal corneal ulcer, particularly following trauma involving vegetative material. Rapid microbiological investigation using Gram and modified acid-fast stains is critical for early and accurate diagnosis. Delays in the recognition or inappropriate use of antifungals may worsen the infection and lead to corneal perforation or the need for therapeutic keratoplasty, underscoring the importance of timely suspicion and targeted therapy. Prompt initiation of topical amikacin therapy can preserve vision, minimise corneal scarring, and significantly reduce patient morbidity.
